# Supplementary material for: Causal interplay between lactose intolerance and gut microbiota: a combined bidirectional Mendelian randomization and in vivo validation study
Source: Front Nutr. 2026 Jun 1;13:1803337. doi: 10.3389/fnut.2026.1803337 (PMC13265576; doi:10.3389/fnut.2026.1803337)
Supplement: Supplementary file 1 [file Data_Sheet_1.zip › supplementary materials/Forward/forest plot/ebi-a-GCST90027820.finngen_R12_E4_LACTONAS.pdf]

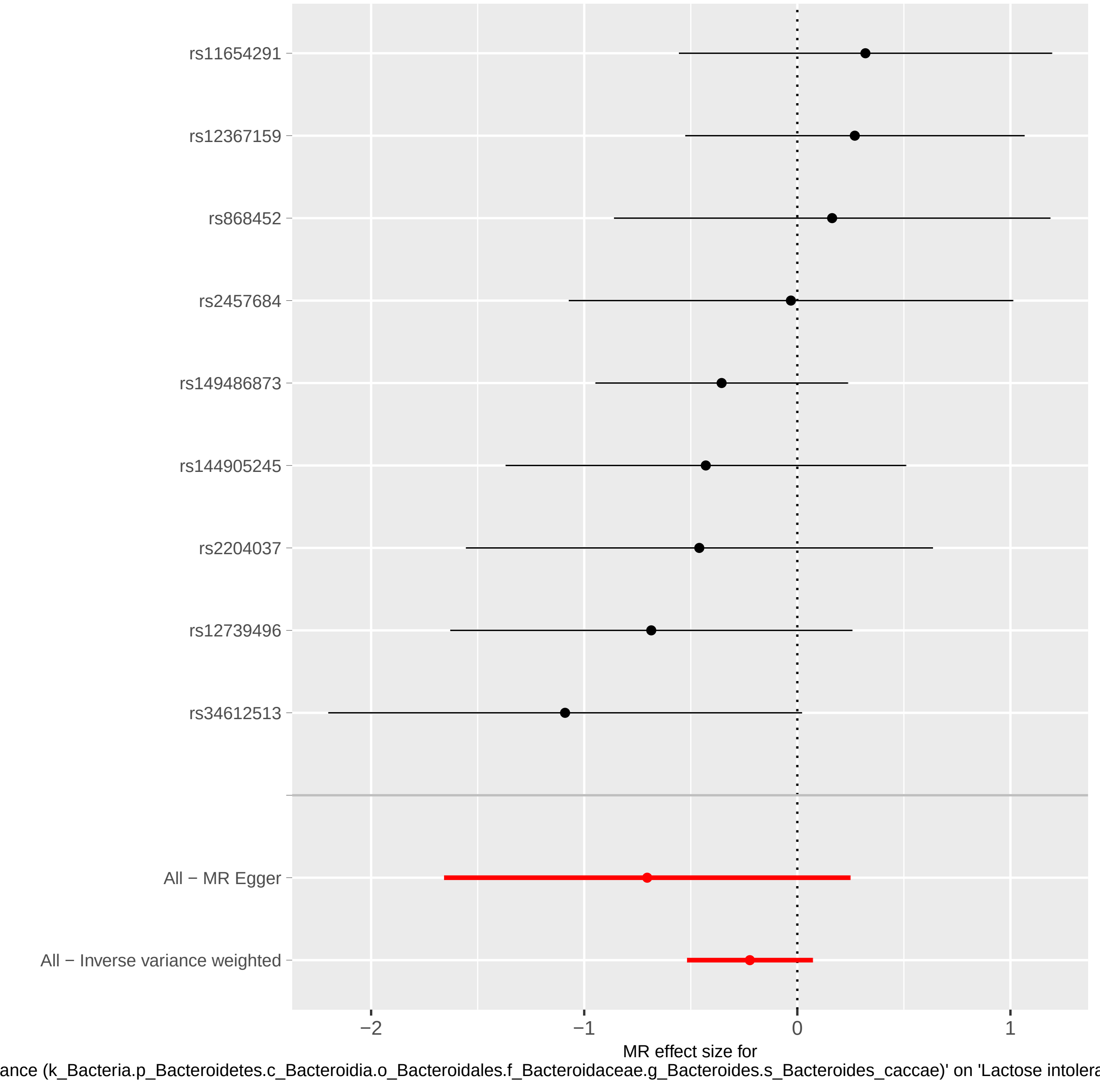

rs11654291
rs12367159
rs868452
rs2457684
rs149486873
rs144905245
rs2204037
rs12739496
rs34612513
All − MR Egger
All − Inverse variance weighted
−2
−1
0
1
MR effect size for
ance (k_Bacteria.p_Bacteroidetes.c_Bacteroidia.o_Bacteroidales.f_Bacteroidaceae.g_Bacteroides.s_Bacteroides_caccae)' on 'Lactose intolera
